# Improving communication between staff and disabled children in hospital wards: testing the feasibility of a training intervention developed through intervention mapping

**DOI:** 10.1136/bmjpo-2017-000103

**Published:** 2017-09-11

**Authors:** Rebecca Gumm, Eleanor Thomas, Claire Lloyd, Helen Hambly, Richard Tomlinson, Stuart Logan, Christopher Morris

**Affiliations:** 1 Department of Child Health, Royal Devon and Exeter NHS Foundation Trust, Exeter, Devon, UK; 2 PenCRU (Peninsula Cerebra Research Unit) and PenCLAHRC, Institute of Health Research, University of Exeter Medical School, Exeter, UK

**Keywords:** disabled children, parents, staff, communication, training

## Abstract

**Objective:**

To develop and test the feasibility of a novel parent-inspired training intervention for hospital ward staff to improve communication with disabled children when inpatients.

**Design:**

Training content and delivery strategies were informed by the iterative process of Intervention Mapping and developed in collaboration with parents of disabled children.

**Setting:**

UK University Hospital children's ward.

**Subjects:**

80 medical, nursing, allied health professionals, clerical and housekeeping staff on a children's ward.

**Methods:**

Themes identified in previous qualitative research formed the basis of the training. Learning objectives included prioritising communication, cultivating empathy, improving knowledge and developing confidence. Participant feedback was used to refine content and delivery. Intervention documentation adheres to the Template for Intervention Description and Replication checklist.

**Results:**

Highlighting mandated National Health Service policies and involving the hospital Patient and Carer Experience Group facilitated management support for the training. Eighty staff participated in one of four 1-hour sessions. A paediatric registrar and nurse delivered sessions to mixed groups of staff. General feedback was very positive. The intervention, fully documented in a manual, includes videos of parent carers discussing hospital experiences, interactive tasks, small group discussion, personal reflection and intention planning. Generic and local resources were provided.

**Conclusion:**

It was feasible to deliver this new communication training to hospital ward staff and it was positively received. Early feedback was encouraging and indicates a commitment to behaviour change. Further piloting is required to establish the transferability of the intervention to other hospitals, followed by consideration of downstream markers to evaluate the effects on disabled children's inpatient experience. Organisational and cultural change is required to support individual behaviour change.

**What is already known on this topic?**

- Disabled children are admitted to hospital more often than other children.
- Research suggests that disabled children’s experience as inpatients is not always optimal and identifies communication between staff, children and families as a key issue.
- Improving children’s experience of healthcare is a priority for the National Health Service.

**What this study hopes to add?**

- We describe development of a novel training using the intervention mapping approach.
- The training comprises videos of parent carers discussing hospital experiences, interactive tasks, small group discussion, personal reflection and intention planning.
- The training was feasible and is documented in a manual to enable replication.

## Introduction

Disabled children should be consulted about their care and decisions that affect them, a right asserted by The UN Conventions on the Rights of the Child^1^ and the Rights of Persons with Disabilities.^2^ The UK is a signatory to both conventions and National Health Service (NHS) policy affirms that children have the right to be treated with respect and enabled to make decisions about their healthcare.^3^ Despite legislation and policy at international and national level to promote equitable healthcare, the practical reality is that poorer experiences are still reported for disabled children.^4^ This is particularly evident with regards to disabled children and their involvement in decision-making processes for their own healthcare and well-being. The NHS has committed to improve patient experience of care through the NHS Outcomes Framework and identified responsiveness to inpatients’ needs as an indicator to evaluate improvement.^5^

Disabled children are more often admitted to hospital than other children.^6 7^ Communication in hospital can be particularly challenging for children with learning disabilities,^8^ and those who use augmentative and alternative communication.^9^ It has been reported that parents often feel unable to leave such children because of concerns about communication.^10–13^ When communication is poor, children and parent carers may not understand their choices and have inadequate opportunity to engage in decision-making.

Synthesis of qualitative research on the experience of disabled children as inpatients suggested that communication mediates many aspects of their experience, but is often inadequate in practice.^14^ Communication was found to be an overarching theme, with impact on other factors and influenced the hospital experience as a whole. Good communication can help to alleviate adverse emotional states, contribute to a more positive perception of the environment and improve confidence in staff. Further qualitative research with parents and ward staff identified key barriers and facilitators to good communication. Barriers included time pressures and the low priority given to communication; facilitators were making time to build a rapport with a child, previous experience of working with children and a family-centred outlook.^15^ The professional participants in our earlier qualitative study clearly expressed awareness and personal frustration with failing to meet children’s communication needs. Some parents felt that their knowledge and experience of their child was not always considered or valued, which has been highlighted in previous studies.^16^ Our findings were similar to those of Oulton *et al*^8^ which emphasised that training in caring for children with learning disabilities was a key factor in taking an individualised approach to inpatient care.^14^

The UK Department of Health Education Outcomes Framework includes five domains, one of which is ‘NHS Values and Behaviours’ and refers to healthcare staff developing values and behaviours, through training, to enhance the quality of the patient experience.^17^ This is a mandate for the provision of relevant training for the existing workforce.

This paper describes research to develop a training package for staff in partnership with parent carers and ward staff called ‘Improving Inpatient Experiences for Disabled Children’, to test the feasibility of delivery in a ward setting and to gauge the utility of such an intervention to staff.

## Methods

### Stakeholder involvement

The research had a strong ethos of public involvement. We report this involvement using GRIPP2 guidance.^18^ Six parents of children with neurodisability from the PenCRU Family Faculty collaborated at various times to develop the training (www.pencru.org/getinvolved/ourfamilyfaculty). Financial acknowledgement of their time and travel expenses were reimbursed. Parent carers suggested the topic, helped design the training, suggested and facilitated invitation to the hospital Patient and Carer Experience Group, recorded their experiences for the video content and participated in meetings to reflect on the training with the facilitator. Parents are also involved in sharing the findings. The involvement of parent carers profoundly influenced the content of the training to deliver family experiences and messages. We did not have resources to plan for the meaningful involvement of young people; involving disabled young people would be desirable in future work. There was little ethnic diversity among the parents we work with; accounting for cultural differences might add another dimension to the training. Paediatricians and nurses were represented on the team and other clinicians were consulted about the design of the intervention.

### Ethics statement

The Royal Devon and Exeter NHS Foundation Trust Research and Development Office approved the study. The Health Research Authority does not require ethics approval for studies involving NHS staff as research participants by virtue of their professional role (www.hra.nhs.uk/resources/before-you-apply/research-requiring-nhs-rd-review-but-not-ethical-review).

### Theoretical underpinning

The process of developing the training was informed by intervention mapping.^19^ Intervention mapping includes six iterative steps for developing and evaluating health interventions (table 1). This study describes the use of the first four steps to design the intervention. We took account of recommendations from the National Institute for Health and Care Excellence (NICE) guidance on behaviour change^20^ and the Template for Intervention Description and Replication.^21^

**Table 1:** Intervention mapping framework

Products	Tasks
Step 1 Needs assessment	Establish a stakeholder group Conduct needs assessment Assess capacity Determine programme outcomes
Step 2 Programme objective matrices	State expected changes in behaviour and environment State performance objectives Specify modifiable determinants Create a logic model of change
Step 3 Theory-based methods and practical strategies	Review programme ideas with representative participants Identify relevant theories Choose programme methods Select or design strategies appropriate to change objectives
Step 4 Programme	Consult intended participants and implementers Create programme scope, sequence and resources list Develop design documents Review available programme materials Draft programme materials Pretest programme materials with target group and implementers. Produce materials and protocols
Step 5 Adoption and Implementation Plan	Identify potential adopters and users Specify adoption, implementation and sustainability performance objectives Specify determinants and create a matrix of change objectives Select methods and strategies Design interventions for adoption and implementation
Step 6 Evaluation plan	Develop evaluation model Develop indicators and measures Specify evaluations designs Write an evaluation plan

### Step 1: Needs assessment

The first step in intervention mapping involves assessing the need for an intervention. The research was initiated by a parent approaching the researchers following a difficult inpatient experience with their disabled child. A structured review confirmed that the inpatient experience of disabled children is not always optimal and that communication is a key determinant of inpatient experience.^14^ A qualitative study was undertaken to explore the experiences of families of disabled children and ward staff and to gain a fuller understanding about the concerns, skills and resources influencing communication. Fifteen parents and 25 ward staff took part in semistructured interviews or focus groups. Difficulty was experienced in recruiting children and evaluating their experiences, despite considerable efforts. Thematic analysis of the interviews and focus groups identified barriers and facilitators to effective communication on children’s wards.^15^

There was no formal-specific training in the hospital focusing on communication or disability. A consultation meeting with staff representative of those requiring training informed the needs assessment to take account of the social and environmental context of the intervention. This explored interest in the training topic, practical considerations that might influence participation.

### Step 2: Identifying outcomes and change objectives

**Figure 1:**
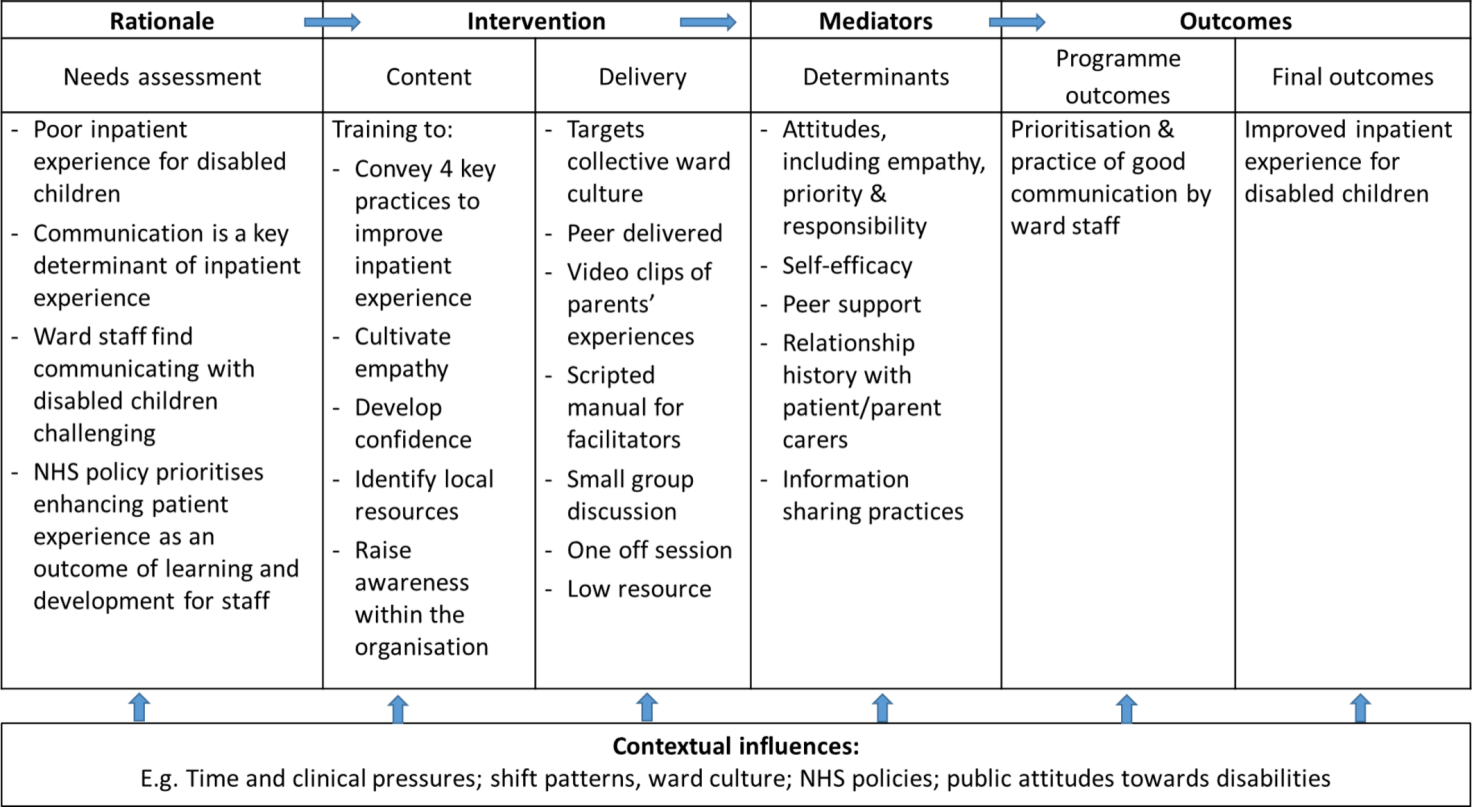
Logic model of the training intervention and outcomes. NHS, National Health Service.

The second step involved considering the objectives of training and creating a logic model of change (figure 1). The ultimate outcome is to improve the inpatient experience of disabled children through enhanced ward staff communication with disabled children and their parent carers. However, this outcome is dependent on many intermediary variables. The more proximal objectives involve who and what needs to change in order to achieve this.

The intended participants were staff who interact with children admitted to the ward. The child’s journey through the ward was considered, including all interactions beyond medical and nursing care; for example, from first booking in with the ward administrator, meal times and meeting ward housekeeping staff. The target behaviour change was that ward staff prioritise and practice good communication with disabled children. Based on our needs assessment and consultation with parents in our advisory group four key practices were identified:

1. Ask the parent or carer for advice about how to communicate with their child.
2. Identify how a child communicates yes and no.
3. Communicate directly with a child when appropriate.
4. Feel comfortable to admit when you don’t know the best way to communicate.

It was evident that for the intervention to succeed, organisational and cultural changes were required to support individual behaviour change. A key contextual factor was for managers to recognise and prioritise the need for training and enable staff attendance.

### Step 3: theory-based methods and practical strategies

The third stage of intervention mapping is to select practical methods and strategies consistent with behaviour change theories. With reference to the NICE guidance, the theories selected as being most appropriate were the Theory of Planned Behaviour^22^ and Bandura’s construct of self-efficacy.^23^ Azjen’s theory states that intention is the main determinant of action, this is predicted by attitude, subjective norm and perceived behavioural control. In Bandura’s construct, self-efficacy is the belief in one’s ability to succeed in specific situations. Table 2 lists the learning objectives for ward staff and how each links with behaviour change concepts and the content of the training.

**Table 2:** Behaviour change concepts mapped to training content

Learning objectives	Behaviour change concepts	Training content
To understand the impact of communication behaviours on disabled children	Outcome expectancies and positive attitude Personal and moral norms	Parent video’s describing their child’s experience and how this could have been improved Inclusion of a positive experience Handouts including research findings
To be motivated to change behaviour	Personal relevance Self-efficacy	Interactive tasks appropriate to role, small group discussion of personal experience Parent videos
To develop empathy	Personal and moral norms	Opportunity for personal and small group reflection Practical exercises Parent videos
To feel capable of behaviour change	Self-efficacy Prompt/cue	Four key practices, reinforced throughout training Basic awareness of some communication aids Signposting to local resources and policies Poster of four key practices displayed on ward
To make a commitment to change	Intention formation Concrete plans	Opportunity to document how the training will change personal practice
To feel supported by the organisation in changing behaviour	Knowing and utilising existing processes and service models	‘Local slot’: highlighting local policies and useful resources

Common principles of adult learning were applied, for example, the concept of experiential learning and learning as a social activity. Continuous Professional Development tends to be more effective when time is allocated to reflect on learning and where organisational support is provided to facilitate change.^24^ This supports the importance of the secondary stream of the intervention; cultural and organisational change to support and maintain individual behaviour change.

### Step 4: Programme development

This step produces the content and delivery of the intervention. Our consultation with ward staff suggested (1) it should not last more than 1 hour; (2) it should not be ‘mandatory training’ as this may mean some people attend reluctantly and (3) a face-to-face group session was preferred to online learning.

It was agreed with the advisory group that in order for the training to be sustainable and reproducible, videos of parents would be used to deliver key messages rather than parent facilitators. The facilitators would be local staff; in this instance the sessions were delivered by a paediatric registrar and a paediatric nurse with experience of working with disabled children. It was perceived that a nurse cofacilitator made the training more accessible to nurses who comprise the majority of ward staff.

Personal and external factors influencing running sessions successfully were identified; this informed development of delivery strategies for the training (table 3). Advertising strategies were considered to ensure that all ward staff were made aware of the training. Face-to-face recruitment on the ward promoted discussion of the training and peer support to attend. Discussion with ward managers from different disciplines supported staff in attending. For example, an agreement was reached for nursing staff to use their half-hour lunch break and be given an additional half hour to enable them to attend the full-hour session.

**Table 3:** Strategies for delivery objectives

Delivery objectives	Personal factors	External factors	Strategies
Raised awareness at organisational level with ‘buy in’ to cultural change	Perceived importance of the need for training	Competing interests and priorities	Meeting with the hospital Patient Carer Experience Group Highlighting statutory requirements Reviewing policies and strategies at children’s ward business meeting
Everyone working on the ward are able to attend training	Knowledge of training sessions	Allocation of time to attend (behavioural control) Accessibility of site Duration of training and timing in day	Meeting with senior staff from all disciplines to agree permission to attend Identifying a suitable site Delivering the training at acceptable timings and duration to allow equity of access
Everyone working on the ward attends training	Confidence to attend Motivation to attend	Modelling by peers who have attended (subjective norms)	Visiting the ward and speaking to staff about the training Signing up peers to attend together Providing lunch as a motivator Identifying key figures and encouraging them to attend

The training included a warm up activity to engage participants, parent videos, interactive tasks and small group discussion. Time was allocated at the end for personal reflection, to document a commitment to behaviour change and to provide feedback. Participants were asked for numerical scores for training elements and to identify two positive points, two areas for improvement and how the session was likely to change their practice. After each session the facilitators reviewed the feedback and proposed changes together, this was also discussed with the advisory group.

## Results

Eighty participants attended one of four sessions at the Royal Devon and Exeter NHS Foundation Trust, which is a UK University Hospital. All sessions were attended by various staff (table 4). Allied health professionals included physiotherapists, play therapists, dieticians, a pharmacist and a speech and language therapist. Non-clinical staff included housekeeping and catering staff, administrators, teachers and chaplain. A notable feedback comment was that ‘it felt very powerful to have such a cross section of staff and hierarchy all working at the same task’.

**Table 4:** Roles of participants attending training

Profession	Number of participants	Roles represented
Medical	34	Seven consultants, 20 junior doctors, seven medical students
Nursing	25	Four senior nurses, four specialty (epilepsy, oncology), 10 ward nurses, five nursing students, two nursing assistants
Allied health professionals	9	Three physiotherapists, two play therapists, two dieticians, one pharmacist, one speech and language therapist
Ward- non-clinical	12	Six housekeeping and catering staff, two ward administrators, 1 chaplain and three teachers

The first session was attended by 26 people, including some unbooked participants. Subsequent sessions were strictly limited to 20. One session had fewer numbers at short notice due to unexpected clinical workload and staff capacity. Participant feedback was very positive, with high satisfaction scores for all areas. Comments in response to asking for positive points indicated that the videos and practical strategies designed to develop empathy and understand the impact of communication behaviours on disabled children had been successful. Comments in response to the question ‘How will you change your practice?’ indicated that participants were willing to make a commitment to change, felt capable of change and were assimilating key messages (box 1).

Box 1 **Examples of feedback**

**Positive points**

‘Easy understanding by watching interviews’

‘Really improved the level of understanding of how children can feel in some situations’

‘Sharing experiences was helpful’

‘Practical exercises were enlightening’

**Areas for improvement**

‘More discussion’

‘Focus less on problems’

‘Some slides text heavy’

‘Room too hot and crowded’

**How will you change your practice?**

‘Considering the patient as an individual’

‘Feel more confident to just ask’

‘Take more time to think’

‘More techniques on how to communicate’

Feedback on areas for improvement included allowing more time for discussion and highlighting parents’ positive and negative experiences. This resulted in the inclusion of a positive parent experience video and distributing background information prior to the session rather than during it, to allow more discussion time. One suggestion was for prompts and reminders to be displayed on the ward. We worked with young people with learning disability at a local further education college to create a poster that provides ‘4 Top Tips’ to improve communication with disabled children.

## Discussion

The training was well received in the context of a university hospital paediatric ward. The number of staff attending and the breadth of roles represented reflect successful recruitment strategies and a high level of interest in improving ward communication with disabled children. Strategies to raise awareness with ‘buy in’ to cultural change at management level were an essential part of the intervention. The commitment to wider cultural change was underpinned by the involvement of stakeholder groups and promotion at ward and hospital level meetings.

The iterative approach of intervention mapping provided a structure to consider the complexity of personal and external determinants that influence behaviour at an individual and organisational level. Consultation provided guiding principles for an acceptable delivery model for training; feedback after sessions led to more focused content and staff feedback indicates provisional willingness and motivation to change behaviour. The intervention mapping process was useful as an approach, however, we found applying it in practice to be time-consuming as it required granular levels of analysis in steps 2 and 3 to define objectives. We followed the approach insofar as it was possible within the limitations of time and resources available. An alternative approach proposing six steps in intervention development,^25^ may be more practical and be sufficient for the needs of future research when the high degree of rigour required by intervention mapping is not feasible.

A high level of commitment and enthusiasm was required by the paediatric registrar to encourage management support for staff participation. Strategic recruitment of influential staff, seeking validation from credible advocates and peer encouragement through word of mouth were vital to recruitment. This approach relied heavily on understanding the social dynamics of the ward. The commitment to cultural change in the hospital benefited from high-level support from the Parent and Carer Experience Group and seizing opportunities to raise the training at ward meetings. While it is difficult to identify the impact of these individual and local contextual mediators, we suggest that the professional leading these initiatives needs to know the key people in their own organisation.

Despite the relative success of delivering this initiative in one hospital, we accept the need for replication in other hospitals, and formal evaluation of the effectiveness of the intervention to actually improve children’s experience of care as inpatients. The transferability of the intervention to other hospitals will help to identify which intervention ingredients can influence organisational and cultural change on a broader scale. The hospital ward is a complex environment with a host of interacting variables. The premise of the logic model underpinning the intervention is that delivering training will lead to communication behaviour change in staff and that this will improve the disabled children’s experience of care as inpatients. This ultimate outcome could be measured using questionnaires developed to measure children’s experience as inpatients.^26 27^ However, before proceeding to such a study, we need to refine the intervention content and be confident in the delivery strategies that it is feasible for the training to be delivered in hospital children’s wards.

Our training was designed to specifically address challenges to communication that arise on a paediatric ward. The group learning resources offered by disability matters are a potentially more comprehensive means to address the learning needs of professionals working with disabled children and young people across different settings.^28^ A Danish study incorporated communication skills into clinical practice using face-to-face methods in a 3-day course. We suggest that this time commitment is unlikely to be realistic in many acute settings.^29^ A strength of this training package is that it was designed purposefully to be short, sustainable and deliverable with minimal resources.

A report by the Care Quality Commission (CQC) in England highlighted inequalities of inpatient experience for children with physical, learning or mental health needs.^30^ There was a call for action by representatives of the CQC, NHS England, professional bodies (RCPCH) and third sector advocacy groups (Young Minds). The importance of good communication was emphasised. These inequalities have persisted despite international and national legislation and ground level hospital policies and procedures. This agenda can be lost during the day-to-day ward pressures, but this is not acceptable. This intervention provides a low cost, fixed time, minimal resource option to raise awareness at organisational level and provides staff with training that is based on parental and child experiences, to motivate behaviour change. We have shown that it is feasible to deliver and that it was well received by staff. Early feedback was encouraging and indicated a real desire among staff to improve their communication skills. The next stage will be to test the transferability to other settings and to formally evaluate its impact. We believe that there is much potential for this intervention to improve the hospital inpatient experience of disabled children.
